# Dietary nitrate supplementation very slightly mitigates the oxidative stress induced by high-intensity training performed in normobaric hypoxia

**DOI:** 10.5114/biolsport.2025.139851

**Published:** 2024-11-18

**Authors:** Ana Sousa, Marie Chambion-Diaz, Vincent Pialoux, Romain Carin, João Luís Viana, Jaime Milheiro, Víctor Machado Reis, Grégoire Millet

**Affiliations:** 1 Research Center in Sports Sciences, Health Sciences and Human Development – CIDESD, University of Maia, UMaia, Maia, Portugal; 2 Laboratoire Interuniversitaire de Biologie de la Motricité, Univ Lyon, University Claude Bernard Lyon 1, Villeurbanne, France; 3 Institut Universitaire de France, Paris, France; 4 CMEP, Exercise Medical Center Laboratory, Porto, Portugal; 5 Research Center for Sports, Exercise and Human Development, University of Trás-os-Montes e Alto Douro, Vila Real, Portugal; 6 Institute of Sport Sciences, University of Lausanne, Lausanne, Switzerland

**Keywords:** Nitrate, Hypoxia, Oxidative stress, HIIT, Supplementation

## Abstract

Oxidative stress is augmented under hypoxic environments, which may be attenuated with antioxidant supplementation. We investigated the effects of dietary nitrate (NO_3_−) supplementation combined with high-intensity training performed under hypoxic conditions on antioxidant/pro-oxidant balance. Thirty trained participants were assigned to one of three groups – HNO: hypoxia (13% F_i_O_2_) + NO_3_−; HPL: hypoxia + placebo; CON: normoxia (20.9% F_i_O_2_) + placebo – while performing 12 cycling high-intensity interval training (HIIT) sessions during a 4-week period (3 sessions/week). Before and after the intervention, venous blood samples were collected and a time to exhaustion test (T_lim_) was performed (with vastus lateralis changes in local O_2_ saturation: SmO_2_ monitoring). Glutathione peroxidase (GPX) activity increased in CON (p = 0.017, ~20%) and superoxide dismutase (SOD), catalase and ferric-reducing antioxidant power (FRAP) did not change in any group. Malondialdehydes (MDA) increased in both HNO (p = 0.001, ~60%) and CON (p = 0.023, ~30%) but not in HPL. Advanced oxidation protein products (AOPP), uric acid, and myeloperoxidase activity were not modified by the protocol. Only the CON group recovered faster after the T_lim_ test (SmO_2recovery_: p = 0.0003, ~58%). Hypoxic exposure during high-intensity training blunted the increase in GPX and MDA after the intervention period. However, the effects of NO_3_− supplementation seem to very slightly mitigate the detrimental effect of performing high-intensity training under hypoxic conditions.

## INTRODUCTION

Although many hypoxic training methods exist, the majority of endurance athletes still utilize altitude training with prolonged exposure to moderate hypoxia (living high, training high) [1]. However, more and more elite athletes who need to repeat high intense efforts during competition include intermittent hypoxic training methods in their training, and, more specifically, high-intensity interval training in hypoxia (HIIT).

Such intermittent hypoxic training has been shown to augment oxidative stress compared to similar training done in normoxia [2]. Indeed, aggravation of oxidative damage through unbalanced DNA strand breakage [3], increase in lipid peroxidation [4] and protein oxidation have been reported under exercise in hypoxic conditions. Although all mechanisms underlying this unnecessary oxidative stress are not entirely clear, they lead to reduced redox potential within the mitochondria as well as increased catecholamine production and activation of the xanthine oxidase pathway (see [2] for more details). This could also be the consequence, not only of the increased activity of ROS generated, but also of the decreased activity of antioxidant systems [5].

Antioxidant supplementation can have beneficial effects in attenuating and/or preventing oxidative damage associated with exercise in hypoxia. Nitric oxide (NO), as an important antioxidant agent that suppresses the formation of free radicals [9], through the NO uncoupling pathway, would be a worthy intervention. However, its bio-availability can be enhanced through an alternative pathway involving sequential reduction of nitrate (NO_3_−) to nitrite (NO_2_−) and further to NO. The latter is independent of oxygen [6], and therefore augmented in hypoxic conditions. Reducing ROS formation is expected to be beneficial, especially mediated by the oxygen independent NO_3_− – NO_2_− – NO pathway. Therefore, increasing NO bio-availability of this pathway may provide potential ergogenic effects for exercise performed in hypoxia, as the availability of NO has been suggested to influence human acclimatisation to altitude [7]. Not-withstanding this hypothesis, there is little evidence on the influence of NO_3_− supplementation on oxidative stress induced by hypoxic high-intensity exercise. Ashmore et al. [8] studied rats exposed to normobaric hypoxia, reporting that dietary NO_3_− supplementation reduced the levels of oxidative stress markers at rest, suggesting that this strategy may be of benefit to individuals exposed to altitude. Carriker et al. [9] investigated changes in oxidative stress and arterial oxygen saturation (SaO_2_) during exercise in hypobaric hypoxia following acute NO_3_− supplementation in well-trained males and concluded that acute NO_3_− supplementation yielded no beneficial changes in oxidative stress. However, to our knowledge, the influence of NO_3_− supplementation on oxidative stress during a longterm period of high-intensity exercise in hypoxia remains unknown.

Whereas the acute combination of both hypoxia and exercise (of low, moderate and high intensities) clearly augments oxidative stress [2, 10] long-term responses to combined stimuli remain debated. In particular, high-intensity training (work performed above the lactate threshold interspersed by periods of low-intensity exercise or complete rest) has been shown to significantly augment oxidative stress mostly via reduced antioxidant capacity [11, 12]. In contrast with these findings, moderate intensity exercise does not seem to modify antioxidant status or significantly alter redox balance [13]. It must be noted that during these interventions the “living high, training low” model was implemented, and therefore exercise sessions were performed in normoxia. Collectively, this suggests that long-term high-intensity training under hypoxic conditions (training high) may regulate even more the systemic redox balance in humans. Therefore, increasing NO bioavailability, via the NO_3_− – NO_2_− – NO pathway, may provide a key complement in reducing oxidative stress induced by exercise (especially at high intensity) in hypoxia.

The aim of the present study was therefore to analyse the effects of dietary NO_3_− supplementation combined with prolonged high-intensity training performed under normobaric hypoxic conditions on antioxidant/pro-oxidant balance. It was hypothesized that enhancing NO production via the NO_3_− – NO_2_− – NO pathway, by dietary NO_3_− supplementation, would mitigate oxidative stress under hypoxic conditions.

## MATERIALS AND METHODS

### Subjects

Thirty trained, developmental age male subjects (mean ± SD: 54.4 ± 8.2 ml · kg · ^−1^min · ^−1^, 36.2 ± 6.3 yrs., 71.5 ± 8.1 kg and 174.8 ± 6.8 cm for relative maximal oxygen uptake: $\dot{\text{V}}$O_2_max, age, weight, and height, respectively), training 4–5 sessions per week (> 10 years of experience), without differences between groups), volunteered to take part in this study [14]. All subjects signed an informed consent form after they had been informed of all experimental procedures and possible risks associated with the experiments. Ethical approval was obtained by the Ethics Committee of the University of Trás-os-Montes and Alto Douro (Reference 14A/CE/2017) and all procedures complied with the ethics code of the Declaration of Helsinki.

### Intervention

This randomized, single-blind, placebo-controlled, independent group study was conducted in a normobaric hypoxic facility (Porto’s Exercise Medical Center, Portugal: b-Cat). Participants performed (cycle ergometer) 12 high-intensity interval training (HIIT) sessions during a 4-week period (3 sessions/week), while randomly assigned to one of three experimental groups: (i) HNO: high-intensity exercise training sessions in normobaric hypoxia (F_i_O_2_ = ~13%, ~3000 m) with NO_3_− supplement; ii) HPL: high-intensity exercise training sessions in normobaric hypoxia (F_i_O_2_ = ~13%, ~3000 m) with placebo supplement, and iii) CON: high-intensity exercise training sessions in normoxia (F_i_O_2_ = 20.9%) with placebo supplement.

Subjects were instructed to maintain their habitual physical activity level and normal diet but were asked to abstain from the use of any chewing gum and antibacterial mouthwashes products [15]. In the week before (baseline) and after the 4-week period (post-intervention; between the second and third day after the last training session), venous blood samples were collected (in the late afternoon/early evening period) from the median cubital vein and participants performed an exercise transition from rest to severe intensity until exhaustion (T_lim_) on a cycle ergometer (Lode Excalibur Sport, Groningen, The Netherlands).

Each week, participants performed two sessions of short aerobic intervals (HIT: 2 sets of 6 × 1 min at 90%Δ with 1 min active recovery between repetitions and 3 min between sets) and one session of repeated sprint training (RST: 4 sets of 6 × 10 s “all-out”, with 20 s active recovery and 3 min between sets) on a cycle ergometer (Lode Excalibur Sport, Groningen, The Netherlands). All training intensities used were relative to specific p$\dot{\text{V}}$O_2_max (assessed in hypoxia for HNO and HPL, and in normoxia for the CON group). The number of repetitions was increased from 6 (1^st^ and 2^nd^ weeks) to 7 (3^rd^ and 4^th^ weeks) in both HIT and RST sessions (for more details see [16]). Supplements were ingested 2.5–3 h prior to each HIIT session. NO_3_− was administered in the form of beetroot juice containing 400 mg of a powdered standardized beetroot extract (containing 2% NO_3_−, ~8.4 mmol) dissolved in 150 ml of water (Sabeet, Sabinsa Corporation). An equivalent volume of currant juice was served as a control drink.

### Measurements

Time sustained was determined through the T_lim_ test and performed at 80%Δ, as previously reported [17]. The test ended when the cadence could no longer be maintained within 10 rpm of the preferred cadence for > 5 s. Before the test, a standard 5 min warm-up exercise (50% of p$\dot{\text{V}}$O_2_max), followed by a 5 min passive rest, was performed. All tests were performed at the same time of day (± 2 h). During the T_lim_ test, changes in muscle O_2_ saturation (SmO_2_) and in total haemoglobin (THb) were assessed in the capillaries (vastus lateralis muscle) through a near-infrared spectroscopy (NIRS) monitor (Moxy monitor, Fortiori Design, Minnesota, USA), positioned as previously suggested [18], and demonstrated to be valid and reliable to measure SmO_2_ [19].

After venous blood samples were withdrawn, plasma was immediately separated by centrifugation (10 min at 3000 *g*; 4°C). Then samples were stowed in aliquots and immediately stored at -80ºC until analysed for: i) subsequent oxidative stress markers (advanced oxidation protein products: AOPP, malondialdehydes: MDA, nitrotyrosine, ferric-reducing antioxidant power: FRAP, and uric acid: UA), ii) antioxidant enzymes (superoxide dismutase: SOD, catalase, glutathione peroxidase: GPX, and myeloperoxidase: MPO) and, iii) nitric oxide metabolites (NO_3_−, NO_2_− and NOx). All assays on plasma sample were conducted by spectrophotometry.

### Data Collection Procedure

Raw SmO_2_ and THb data were treated using a smooth spline filter to reduce the noise created by movement and data presented every 2 s. Baseline SmO_2_ (SmO_2base_) and baseline THb were computed as a 30-s average while subjects performed 3 min of unloaded baseline pedalling (8 W) at their preferred cadence before the beginning of each test. Minimum SmO_2_ (SmO_2min_) was the lowest 6-s average obtained during each test. Maximum SmO_2_ (SmO_2max_) and maximum THb were the highest 6-s average obtained during each test with the recovery phase included. Average SmO_2_ from 30 to 120 s after the end of each test was used to assess recovery of SmO_2_ (SmO_2recovery_). For each test, baseline SmO_2base_ and SmO_2min_ are expressed as % of SmO_2max_ (relative-SmO_2base_ and relative-SmO_2min_, respectively). Change in SmO_2_ (DSmO_2_) and change in THb DTHb were calculated as the difference between relative SmO_2min_ and relative SmO_2base_ and the difference between maximal and baseline THb [18].

### Blood Samples

Catalase activity in the plasma was determined using H_2_O_2_ as a substrate and formaldehyde as a standard. Catalase activity was determined by the formation rate of formaldehyde induced by the reaction of methanol and H_2_O_2_ using catalase as the enzyme (intra-assay coefficient of variation: CV = 3.1%). GPX activity was determined as the rate of oxidation of NADPH to NADP+ after addition of glutathione reductase (GR), reduced glutathione (GSH) and NADPH, using H_2_O_2_ as a substrate (intra-assay CV = 4.6%). SOD activity was determined by the degree of inhibition of the reaction between superoxide radicals, produced by the hypoxanthine—xanthine oxidase system, and nitroblue tetrazolium (intra-assay CV = 5.6%). MPO activity was measured in plasma by determination of the kinetic absorbance at 653 nm after addition of H_2_O_2_ and 3,3’,5,5’-tetra-methylbenzidine (TMB: intra-assay CV = 5.1%). FRAP concentration was calculated using an aqueous solution of known Fe^2+^ concentration (FeSO_4_, 7H_2_O_2_) as standard at a wavelength of 593 nm (intra-assay CV = 2.9%). The concentration of plasma UA was determined using a commercially available kit. The principle is: uricase acts on uric acid to produce allantoin, CO_2_ and H_2_O_2_. The absorbance is proportional to the uric acid quantity in the sample (intra-assay CV = 0.9%). AOPP assay was calibrated with a chloramine-T solution that absorbs at 340 nm in the presence of potassium iodide. AOPP concentrations were expressed as μmolL^−1^ of chloramine-T equivalents (intra-assay CV = 5.4%). MDA concentration was determined by extracting the pink chromogen with n-butanol and measuring its absorbance at 532 nm by spectrophotometry using 1,1,3,3-tetrae-thoxypropan as standard (intra-assay CV = 2.2%). The metabolites of NO, NO_2_− and NO_3_− were measured using the reagent of Griess, a mixture of sulfanilamide, naphthalene ethylene diamine dihydro-chloride and phosphoric acid. This reagent binds nitrite to form a dye which absorbs at 550 nm. In a second measurement, the NO_3_− reductase was added to the plasma sample in order to convert NO_3_− into nitrite to measure the total amount of nitrites and nitrates (NOx) (intra-assay CV = 3.9, 5.2 and 4.8% for NO_2_−, NO_3_− and NOx, respectively). The plasmatic nitrotyrosine concentration was measured by the ELISA method (enzyme-linked immunosorbent assay: intra-assay CV = 6.8%).

### Statistics

It was considered 10 participants per group for a type I error of 5%, a power of 80%, with statistical significance, and an average population effect size to be detected with probability of 0.5 (G*Power software version 3.1.9.2), considering the time-to-task failure variable at the severe intensity exercise domain. The Shapiro-Wilk test was used to confirm data normality and homogeneity. Data are presented as mean ± SD. Repeated measures analysis of variance (ANOVA) with two factors (group × time) was used to test main and interaction effects for the studied variables. A contrast analysis was used for post-hoc comparisons when an interaction effect was observed (Bon-ferroni test). Magnitudes of standardized effects (partial eta square – r^2^) were determined as follows: small, 0.2–0.5; moderate, 0.5–0.8, and large, > 0.8. All statistical procedures were conducted with SPSS 24.0 and the significance level was set at 5%.

## RESULTS

**FIG. 1:**
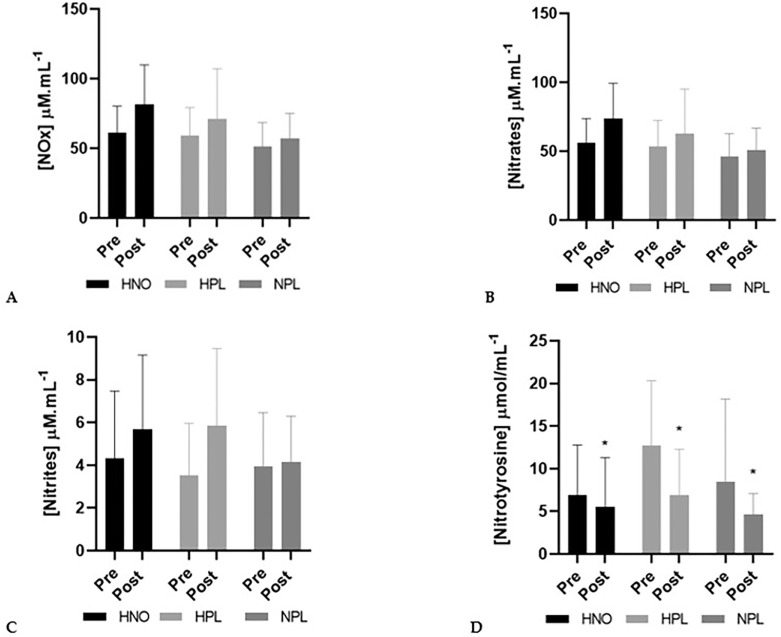
Plasma concentration (mean + SD) of nitrate reductase (NOx: panel A), nitrates (panel B), nitrites (panel C) and nitrotyrosine (panel D) in pre-intervention (1 week before the beginning of training and supplemen-tation) and in post-intervention (1 week after the end of training but following supplementation) for high-intensity exercise training sessions performed in hypoxia with NO3-(HNO: black), in hypoxia with placebo (HPL: light grey) and in normoxia with placebo (CON: dark grey). Significant differences: * Compared to Pre (p<0.05)

NOx increased (+22%) between pre- and post-intervention periods, though not significantly (time effect: *p* = 0.06). Also, plasmatic nitrates (+21%) and nitrites (+33%) increased non-significantly (time effect: *p* = 0.07 and *p* = 0.09 for nitrates and nitrites, respectively). Only nitrotyrosine significantly decreased (time effect: *p* = 0.04) from pre- to post-intervention, regardless of the group (-22% in the HNO group, -41% in the HPL group and -45% in the control group) (Figure 1).

**FIG. 2:**
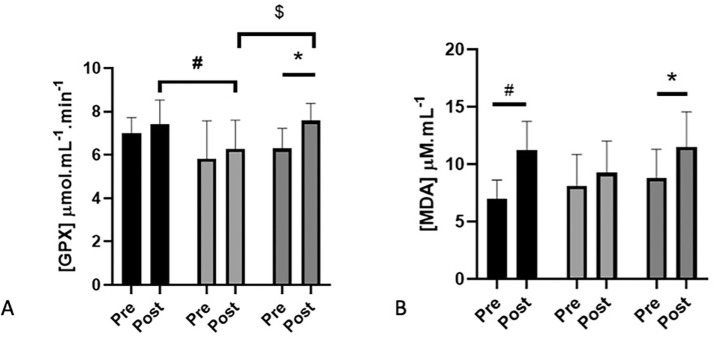
Plasma activity (mean + SD) of glutathione peroxidase enzyme (GPX: panel A) and plasma concentration of malondialdehyde (MDA: panel B) in pre-intervention (1 week before the beginning of training and supplementation) and in post-intervention (1 week after the end of training but following supplementation) for high-intensity exercise training sessions performed in hypoxia with NO_3-_ (HNO: black), in hypoxia with placebo (HPL: light grey) and in normoxia with placebo (CON: dark grey). Significant differences: * Compared to Pre (p<0.05); # HNO post > HPL post (p<0.05); $ CON post > HPL post (p<0.05)

There was a time effect for GPX (+12%, *p* = 0.025), increasing but only in CON (*p* = 0.017, 20%) and not in the HNO and HPL groups. In addition, at post-intervention, GPX activity in plasma was lower for HPL compared to HNO (*p* = 0.04) and CON (*p* = 0.01) groups (Figure 2A). In contrast, SOD, catalase and FRAP concentration were not modified between pre- and post-intervention for the 3 groups (Table 1).

**TABLE 1:** Mean ± SD superoxide dismutase activity (SOD), catalase concentration and ferric reducing ability power (FRAP), advanced oxidation protein product (AOPP), myeloperoxidase (MPO) and uric acid activity at pre-intervention (1 week before the beginning of training and supplementation) and post-intervention (1 week after the end of training but following supplementation) for high-intensity exercise training sessions performed in hypoxia with NO_3_− (HNO), in hypoxia with placebo (HPL) and in normoxia with placebo (CON)

	HNO		HPL		CON	

	Pre	Post	Pre	Post	Pre	Post
SOD (μmol · mL^−1^ · min^−1^)	1.95 ± 0.59	2.26 ± 0.46	1.93 ± 0.61	1.83 ± 0.56	1.88 ± 0.71	2.06 ± 0.48
Catalase (μmol · mL^−1^)	2.20 ± 0.47	2.80 ± 1.48	2.78 ± 0.87	2.37 ± 0.82	2.35 ± 0.91	2.12 ± 0.89
FRAP (mmol · L^−1^)	583 ± 87	516 ± 162	503 ± 166	535 ± 69	463 ± 137	410 ± 154
AOPP (μmol · mL^−1^)	122.4 ± 63.2	152.3 ± 110.7	141.0 ± 72.1	151.2 ± 59.5	102.8 ± 90.1	109.1 ± 71.9
MPO (mmol · L^−1^ · min^−1^)	12.67 ± 5.09	15.37 ± 4.01	11.43 ± 5.00	15.74 ± 5.65	16.01 ± 6.65	12.54 ± 5.56
Uric acid (μmol · L^−1^)	471 ± 101	513 ± 102	496 ± 132	580 ± 80	479 ± 104	499 ± 147

There was a time effect for MDA (+34%, *p* = 0.0003). MDA increased in HNO (+60%; *p* = 0.001) and in CON (+30%; *p* = 0.023) but not in the HPL group (Figure 2B). Conversely, AOPP, uric acid, and myeloperoxidase activity were not modified by the protocol whatever the group (Table 1).

SmO_2recovery_ decreased (20%) from pre- to post-training (time effect: *p* = 0.01). However, the decrease was significant only in CON (58%; *p* = 0.0003) but not in HNO or HPL. In addition, at post-in-tervention, SmO_2recovery_ was higher in HNO (*p* = 0.001) and HPL (*p* = 0.0006) than in the CON group (group effect: *p* = 0.02; interaction effect: *p* = 0.01) (Table 2). At post-intervention, SmO_2base_ (25%; *p* = 0.007) and relative SmO_2base_ (22%; *p* = 0.002) were lower in HPL compared to the CON group. This latter parameter was also lower in HPL (8%; *p* = 0.03) compared to the HNO group. SmO_2min_ (61%; *p* = 0.003) and relative SmO_2min_ (60%; *p* = 0.04) were lower in HNO compared to the CON group. In addition, DSmO_2_ was also lower in HNO compared to the HPL group (15%; *p* = 0.04) (Table 2).

**TABLE 2:** Mean ± SD values for oxygen saturation parameters obtained during the Tlim at pre- (1 week before the beginning of training and supplementation) and post-intervention (1 week after the end of training but following supplementation) for high-intensity exercise training sessions performed in hypoxia with NO_3_− (HNO), in hypoxia with placebo (HPL) and in normoxia with placebo (CON)

	HNO		HPL		CON	

	Pre	Post	Pre	Post	Pre	Post
SmO_2base_ (%)	71.44 ± 9.81	69.00 ± 11.36	62.00 ± 7.30	64.30 ± 8.77^##^	71.70 ± 7.10	75.44 ± 6.31
SmO_2min_ (%)	5.44 ± 5.05	4.67 ± 3.35^##^	5.14 ± 8.32	8.10 ± 9.72	8.20 ± 8.36	12.78 ± 9.63
SmO_2max_ (%)	80.78 ± 5.36	77.33 ± 8.20	78.71 ± 4.03	78.50 ± 5.50	82.60 ± 3.03	82.00 ± 6.61
SmO2recovery (%)	72.82 ± 8.43	68.49 ± 13.78^##^	69.72 ± 7.50	69.68 ± 8.76^##^	71.83 ± 7.17	42.19 ± 34.09^*^
Relative-SmO_2base_ (% of SmO_2max_)	88.17 ± 7.75	88.80 ± 6.96^# $^	78.69 ± 7.52	81.67 ± 6.49^##^	86.73 ± 6.90	92.33 ± 8.14
Relative-SmO_2min_ (% of SmO_2max_)	6.58 ± 5.86	6.08 ± 4.24^#^	6.63 ± 10.82	10.17 ± 12.28	9.95 ± 9.92	15.16 ± 11.25
DSmO_2_ (%)	-81.59 ± 8.37	-82.71 ± 8.22^$^	-72.06 ± 16.37	-71.50 ± 12.49	-76.78 ± 10.03	-77.17 ± 14.41
[DTHb] (AU)	0.12 ± 0.11	-7.86 ± 23.74	0.13 ± 0.11	0.11 ± 0.12	0.22 ± 0.16	0.08 ± 0.20

SmO_2base_, SmO_2min_, SmO_2max_ and SmO_2recovery_: Baseline, minimum, maximum and recovery SmO_2base_, respectively; Relative-SmO_2base_ and Relative-SmO_2min_: SmO_2base_ and SmO_2min_ expressed as % of SmO_2max_, respectively; DSmO_2_ and [DTHb]: change in SmO_2_ and [THb], respectively. Significant differences:

* Compared to Pre (p < 0.5);

# (p < 0.05) and

## (p < 0.01) compared to corresponding control;

$ compared to corresponding HPL (p < 0.05)

## DISCUSSION

The aim of this study was to analyse the effects of a prolonged combination of dietary NO_3_− supplementation with HIIT performed in normobaric hypoxia on antioxidant/pro-oxidant balance in male endurance subjects. It was hypothesized that dietary NO_3_− supplementation would mitigate the detrimental effect of hypoxia on oxidative stress, mainly by enhancing NO production via the NO_3_− – NO_2_− – NO pathway. Our main results showed that hypoxia inhibits the increase in GPX activity as only the CON group showed differences between pre-and post-intervention periods. However, our initial hypothesis that dietary NO_3_− supplementation would mitigate the detrimental effect of hypoxia on oxidative stress was confirmed. It appears that NO_3_− supplementation (in the HNO group) could compensate for the inhibitory effect of hypoxia (observed in HPL group), although hypoxia limited the increase in MDA in response to HIIT.

Our results confirmed that a normoxic HIIT increased GPX activity in plasma as already observed in untrained subjects [20], where-as in our study, the subjects were endurance trained. Hypoxia (with and without NO_3_− supplementation) attenuated this increase in GPX following 4 weeks of HIIT exposure. One may hypothesize that the total metabolic stimulus (exercise demands) of HIIT sessions performed in hypoxia was lower and induced lower mitochondrial ROS production [21, 22]. However, the total energy produced and the distance achieved in HIIT sessions (averaged over the 12 training sessions) were not different between the HNO, HPL and CON groups, suggesting that the relative exercise intensity performed was similar.

Our results of GPX are also contradictory to those showing that acute hypoxia increases oxidative stress and decreases the activity of antioxidant enzymes [23]. Only one study has measured the activity of antioxidant enzymes following a 3-week training program of HIIT in hypoxia [24]. These authors observed an increase in GPX activity from pre- to post-intervention. However, several differences may explain the discrepancy between this study and ours: the athletes were professional, with $\dot{\text{V}}$O_2_max values on average 30% higher than in the present study [24]. On the other hand, although the intensity of the exercise sessions was lower (95% of lactate threshold), the duration of intervals was longer than ours. Consequently, the impact on mitochondrial activity and the resulting radical production was likely stronger (30–40 min of intensive intervals). Finally, Michalczyk et al. [24] measured the GPX activity in red blood cells while we measured it in plasma, which reflects more the systemic pro-antioxidant balance.

Although several previous studies have reported that endurance training in normoxia [20, 25] or in hypoxia [26, 27] induces an improvement in the SOD and catalase activities, we did not observe any significant change for these two antioxidant enzymes. Nevertheless, it is important to emphasize that the work of Miyazaki et al. [20] was conducted with an untrained population, whereas the participants in the present study were endurance trained. Therefore, one may speculate that the activities of these antioxidant enzymes at the beginning of the intervention were already sufficiently high in our subjects compared to untrained subjects, thus contributing to limit their increase [2]. Antioxidant enzymes are higher for endurance athletes vs. sedentary or non-endurance/sprint athletes. In support of this, our results corroborate those of Robertson et al. [25], who did not report a significant improvement in SOD and catalase following HIIT in both normoxia and hypoxia in highly endurance-trained cyclists.

Hypoxia blunted the MDA increase induced by the training intervention (i.e., increase in CON group vs no change in HPL group). This could be a result of lower ROS production in mitochondria in hypoxia during the HIIT sessions. Unlike GPX, it appears that NO_3_− supplementation blunted the inhibitory effect of hypoxia on MDA increase after 4 weeks of HIIT training.

The first hypothesis would be that NO_3_− supplementation could have induced a higher intensity (i.e., power output) performed during HIIT sessions in hypoxia, as proposed by Cocksedge et al. [28]. This would therefore increase the production of ROS mainly from the upregulation of NADPH oxidase 2 and activation of the phospholipase A2 pathway. However, since there were no differences in energy (419.91 ± 52.76 vs. 442.04 ± 66.84 kJ) or distances covered (27999 ± 3576 vs. 29540 ± 4530 m) during the 12 training sessions between HNO and HPL, respectively, this mechanism should not be considered. It is also unlikely that the lipid content and the amount of plasma lipid peroxidation substrate were increased by NO_3_− supplementation. Indeed, such supplementation seems rather to lower plasma triglycerides and cholesterol [29]. Another hypothesis to explain the MDA increase in plasma might be related to the effects of NO_3_− on oxygen availability in muscles. In the HNO group, post-training SmO_2_min was lower than in the other groups (Table 2), suggesting that NO_3_− intake may induce greater vasodilation and therefore a greater oxygen delivery to active muscles in hypoxia [28, 30, 31]. This later mechanism during hypoxic exercise under NO_3_− supplementation would therefore induce greater ROS mitochondrial production and lead to an increase in the production of MDA.

The small effect of NO_3_− supplementation on the oxidative stress and antioxidant markers could be explained by the very modest increase in nitrates in the circulation. It should also be acknowledged that the blood samples were collected a few days after the last supplementation. It was reported previously that there was no additional improvement in exercise tolerance after ingesting beetroot juice containing 16.8 compared with 8.4 mmol NO_3_− over 24 h [32]. In this context, the nitrates dose in our study may therefore be too low (8.4 mmol before each session) to stimulate NO metabolism. The necessary dose of ingested nitrates to significantly increase plasma nitrate concentration, especially chronically, should be higher in endurance-trained athletes (0.07 mmol NO_3_−/kg body weight per day) [33]. This limited increase in plasma nitrate concentrations could be explained by the characteristics of our trained subjects, who usually present greater endothelial NOS (nitric oxide synthase) activity and therefore high endogenous NO production [34]. In addition, trained subjects have higher plasma nitrite concentrations than sedentary or active subjects [35], and also the response to a standard dose of nitrates may be lowered [36]. Finally, recent evidence showed that nitrate supplementation preferentially modified contractile function in type II fibres (vs. type I), which are in lower proportion in endurance athletes, likely explaining the limited physiological response to nitrate supplementation [37].

Regardless of the condition (hypoxia and/or NO_3_− supplementation), the 4 weeks of HIIT training had only minor effects on plasma nitrite levels. Our results confirm those obtained by Dreißigacker et al. [38] showing that high-intensity exercise in normoxia did not induce a significant increase in nitrites in trained cyclists. These authors suggested that nitrite was probably more reduced in NO in erythrocytes during high-intensity than during low-intensity exercises.

Through its ability to produce peroxynitrite by reacting with the superoxide anion, NO could also cause nitrosative stress on biomolecules and increase its end products, such as nitrotyrosine [39]. Nevertheless, we did not observe any significant change in plasmatic nitrotyrosine, either in the NPL and HPL groups, or in the HNO group. It has been observed in highly trained endurance athletes that NO_3_− supplementation containing 8 and 16 mmol of nitrates did not induce a significant increase in plasma peroxynitrite following an incremental and maximal effort on a treadmill [40]. On the other hand, when nitrate supplementation contained 24 mmol of nitrates, these authors observed a significant increase in plasma peroxynitrite. Therefore, the relatively low dose of nitrate (8.4 mmol) ingested before each training session by the participants of the HNO group may partly explain the lack of a significant effect of nitrate supplementation on plasma nitrotyrosine.

We did not detect any significant change in plasma AOPP and uric acid. To our knowledge, no study has measured the effects of such hypoxic training intervention (i.e., living low, training high) on plasma AOPP. In the context of the present study, one may hypothesize that the ROS generated during the training sessions was not sufficient to induce significant protein oxidation, regarding the enzymatic antioxidant capacities of the endurance-trained participants [2]. Finally, in agreement with our results, two recent studies have shown, in endurance-trained athletes, that HIIT training carried out in normoxia or hypoxia did not induce a significant modification of plasma uric acid [24, 40].

One may question the relevance of using time-to-exhaustion exercise since performance time is known as less reliable than for timetrial exercise. First, as reported by Hopkins et al. [41], the constant-power test is not better or worse than a constant-work or constant-duration test. When converted to mean power, constantload tests are more reliable than the other tests. Secondly, by definition, time-to-exhaustion exercise does not require the intensity to be paced. Consequently, this minimizes the potential changes in lactate production and in the oxidative-glycolytic balance of the exercise (known [42] to be one of the most important determinants of the physiological responses, including muscle de-reoxygenation) during high-intensity exercises in hypoxia.

In the present study we adopted an independent group design (not a crossover one), and although we ensured that all groups were blinded for both the intervention (normoxia vs. hypoxia conditions) and the supplementation (NO_3_− vs. placebo) conditions, the fact that some of the participants may have identified the group to which they were allocated may have slightly influenced the results obtained. In addition, it cannot be excluded that a higher daily dose of NO_3_− may have a more pronounced effect. Moreover, since heart rate, lactate or rate of perceived exertion responses are either directly influenced by hypoxia or are irrelevant for RST, the quantification of internal training loads is difficult in the present study. Also, it is important to bear in mind that although the NIRS device used in the present study is a valid and reliable one, there is still a need for further development of this equipment at higher intensities.

## CONCLUSIONS

The present study focused on the effects of high-intensity normobaric hypoxic training associated with NO_3_− supplementation on oxidative stress, antioxidant defence and NO metabolism in endurance subjects. Normobaric hypoxic exposure during HIT and RST sessions blunted the increase in GPX and MDA at the end of the training period. In addition, since the NO_3_− supplementation used in the study had only a modest effect on plasma nitrate and nitrite contents, its effects only very slightly mitigate the detrimental effects of high-intensity training under normobaric hypoxic conditions on oxidative stress and antioxidant markers. Future studies should test a higher dose of NO_3_− to conclude whether NO_3_− supplementation can provide beneficial effects on the oxidative stress-NO metabolism axis in response to high intensity normobaric hypoxic training. Considering the latter, NO_3_− supplementation cannot be currently recommended to mitigate the detrimental effects of high-intensity training under hypoxic conditions on oxidative stress and antioxidant markers.
